# PHYSICAL ACTIVITY TOGETHER FOR COUPLES WITH MILD COGNITIVE IMPAIRMENT: A FEASIBILITY STUDY

**DOI:** 10.1093/geroni/igac059.2961

**Published:** 2022-12-20

**Authors:** Sangwoo Ahn, Sandy Cobb, Scott Crouter, Chung Eun Lee, Joel Anderson

**Affiliations:** University of Tennessee at Knoxville, Knoxville, Tennessee, United States; University of Tennessee at Knoxville, Knoxville, Tennessee, United States; University of Tennessee at Knoxville, Knoxville, Tennessee, United States; Baruch College, New York, New York, United States; University of Tennessee at Knoxville, Knoxville, Tennessee, United States

## Abstract

Spouses often assume the role of informal caregivers for older adults living with mild cognitive impairment (MCI). These couples represent a population with low levels of physical activity, however, there is limited research regarding promotion of physical activity among these dyads. The purpose of this study was to evaluate the feasibility of a dyadic intervention, hypothesized to enhance physical activity via mutual support. We recruited three dyads (mean age: 66 [MCI] and 67 [spouse] years) from a local memory clinic. The dyads were asked to engage in physical activity together for 16 weeks. The weekly intervention consisted of 150 minutes of moderate-to-vigorous physical activity (MVPA) on their own and two lower body strength training sessions per week, one videoconferencing-supervised and one on their own. The adherence rate to the weekly MVPA and strength training was 88% and 83%, respectively. However, the percentage meeting the goal of 150 minutes/week of MVPA as measured using a Fitbit was 31% and 73% among those with MCI and spouses, respectively. There were no issues with the retention rate (100%) or safety; satisfaction scores were high (MCI = 93%, spouses = 98%). Exit interviews revealed three key themes: (1) value of the program (accountability, togetherness), (2) difficulties (differences in physical capacity between spouses), and (3) suggestions to improve the program (incorporating various types of strength training). Our dyadic intervention was feasible to support regular physical activity for older adults living with MCI and their spouses. Future work will incorporate participants’ feedback to optimize the intervention.

